# Explanatory models of hypertension in Guatemala: recognizing the perspectives of patients, family members, health care providers and administrators, and national-level health system stakeholders

**DOI:** 10.1186/s12889-022-14668-7

**Published:** 2022-12-12

**Authors:** Juan Carlos Figueroa, Alejandra Paniagua-Avila, Ingrid Sub Cuc, Sayra Cardona, Manuel Ramirez-Zea, Vilma Irazola, Meredith P. Fort

**Affiliations:** 1 INCAP Research Center for the Prevention of Chronic Diseases, Institute of Nutrition of Central America and Panama, Calzada Roosevelt 6-25 Zona 11, 01011 Guatemala City, Guatemala; 2 Dornsife School of Public Health, Department of Epidemiology and Biostatistics, Drexel University, Urban Health Collaborative, Philadelphia, PA USA; 3 Mailman School of Public Health, Department of Epidemiology, Columbia University, New York City, New York, USA; 4 Department of Native American Studies, University of California, Davis, Davis, CA, USA; 5 Institute for Clinical Effectiveness and Health Policy, Buenos Aires, Argentina; 6 Colorado School of Public Health, Anschutz Medical Campus, Aurora, CO USA

**Keywords:** Qualitative research, Blood pressure, Cardiovascular disease, Hypertension, Rural, Central America, Health behavior

## Abstract

**Background:**

Uncontrolled hypertension is a major public health burden and the most common preventable risk factor for cardiovascular diseases in Guatemala and other low- and middle-income countries. Prior to an initial trial that evaluated a hypertension intervention in rural Guatemala, we collected qualitative information on the needs and knowledge gaps of hypertension care within Guatemala’s public healthcare system. This analysis applied Kleinman’s Explanatory Models of Illness to capture how patients, family members, community-, district-, and provincial-level health care providers and administrators, and national-level health system stakeholders understand hypertension.

**Methods:**

We conducted in-depth interviews with three types of participants: 1) national-level health system stakeholders (*n* = 17), 2) local health providers and administrators from district, and health post levels (25), and 3) patients and family members (19) in the departments of Sololá and Zacapa in Guatemala. All interviews were conducted in Spanish except for 6 Maya-Kaqchikel interviews. We also conducted focus group discussions with auxiliary nurses (3) and patients (3), one in Maya-Tz’utujil and the rest in Spanish. Through framework and matrix analysis, we compared understandings of hypertension by participant type using the Explanatory Model of Illness domains -etiology, symptoms, pathophysiology, course of illness, and treatment.

**Results:**

Health providers and administrators, and patients described hypertension as an illness that spurs from emotional states like sadness, anger, and worry; is inherited and related to advanced age; and produces symptoms that include a weakened body, nerves, pain, and headaches. Patients expressed concerns about hypertension treatment’s long-term consequences, despite trying to comply with treatment. Patients stated that they combine biomedical treatment (when available) with natural remedies (teas and plants). Health providers and administrators and family members stated that once patients feel better, they often disengage from treatment. National-level health system stakeholders referred to lifestyle factors as important causes, considered patients to typically be non-compliant, and identified budget limitations as a key barrier to hypertension care. The three groups of participants identified structural barriers to limited hypertension care (e.g., limited access to healthy food and unaffordability of medications).

**Conclusion:**

As understandings of hypertension vary between types of participants, it is important to describe their similarities and differences considering the role each has in the health system. Considering different perceptions of hypertension will enable better informed program planning and implementation efforts.

## Background

In 2010, estimates indicated that the global age-standardized prevalence of hypertension was 31.3%, and was higher in low and middle-income countries (LMICs) (31.5%) as compared to high-income countries (HIC) (28.5%) [1, 2]. According to the Global Burden of Disease, high systolic blood pressure is one of the five leading risk factors for mortality and disability-adjusted life-years in Central America [3] and available information suggests a high prevalence of risk factors for chronic diseases in Central America [4]. According to the Pan-American Health Organization health indicators, 51% of men and 60% of women in Guatemala are overweight and obese; 37% of adults do not perform enough physical activity; the prevalence of raised blood pressure is 22% in men and 20% in women, and of raised blood glucose/diabetes is 9% in men and 10% in women (estimated data between 2014 and 2016) [5]. Hypertension and relatesd chronic conditions is a notable concern for rural, indigenous people in Guatemala [6], where a high prevalence of non-communicable disease risk factors has been reported [7].

The Guatemalan Ministry of Health (MoH) is the primary public agency providing health care services in the country. The MoH covers 70% of the population and is financed by state tax revenues, loans, international donations, and is complemented by fees received for services [8]. Out-of-pocket costs are high placing a burden on individuals who finance over half of all health expenditures in the country [9] and 70% of medications [8]. The MoH is organized in a network of referral services at the primary, secondary, and tertiary care levels. First level care is supported by health posts; second level is offered by health centers and comprehensive maternity centers, and tertiary level care is provided at hospitals [10]. In health posts, care is provided primarily by auxiliary nurses with less than one year of academic training. The first and second care levels are coordinated by the Sistema Integral de Atención en Salud (SIAS, Comprehensive Health Care System), an institutional structure that focuses on access to services for vulnerable populations, mainly indigenous communities in rural areas [8, 10].

As part of a process to adapt and implement a hypertension control intervention for the Guatemalan public primary health care system [11], we conducted a needs assessment including qualitative data from different geographic areas and levels important to Guatemala’s primary health care system related to hypertension prevention and care [12]. For the needs assessment we used the World Health Organization (WHO)’s building blocks framework to assess the health system’s performance and define priority needs within six different components: service delivery, workforce, information systems, access to essential medicines, financing, and leadership and governance [13]. We captured experiences and perspectives of participants at different levels of the public healthcare system: national-level Ministry of Health staff and health system experts, provincial-, district-, and community-level health providers and administrators, and patients and family members. The breadth of qualitative data collected for the needs assessment allowed us to apply an additional framework to understand different actors’ diverse perspectives about hypertension, which is what we present in this article. We applied Arthur Kleinman’s Explanatory Model of Illness [14] framework to analyze perspectives about hypertension by actor type. Per Kleinman, “medical systems are *both* social and cultural systems. [..] they are not simply systems of meaning and behavioral norms, but those meanings and norms are attached to particular social relationships and institutional settings” [14]. We interpret Guatemala’s MoH as the institutional setting that defines and manages hypertension care, that influences what health providers and administrators can offer, and engages in interactions between public sector providers and patients. However, patients behave within their own cultural system, following behaviors and meanings of care that may differ from those of MoH staff and providers. Understanding the spectrum of explanatory models of hypertension for different actors working within and accessing care from the system is important for tailoring hypertension interventions to local understandings of illness. Different perspectives that are important to understand include: national-level Ministry of Health staff and health system experts, health providers and community members, indigenous and non-indigenous people, and people based in urban and rural areas.

The purpose of this paper is to describe and compare the understandings and perceptions about hypertension between three types of actors within Guatemala’s public health system where the intervention has been implemented [15] in order to continually inform and improve service offerings.

## Methods

### Setting

This study was carried out in Guatemala City and two other Guatemalan departments: Zacapa and Sololá. In total, Guatemala has 22 departments, with a population of 14,9 million and 25 official languages (22 Maya languages, Xinka, Garífuna and Spanish). Fifty six percent of the population identifies as ladino^1^ and 41,7% as Maya. Most of its population (20.2%) lives in the department of Guatemala, where the capital (Guatemala City) is located [16].

Zacapa is composed mostly of ladinos (97.4%) and has a population of 245,374 people [16]. Its indigenous populations, particularly Ch’orti’, are today a minority due to infectious diseases during colonization, exploitation and expropriation of land, displacement, forced labor, and droughts [17, 18]. Since colonial times, it has been an important region for cattle raising [17, 19].

Sololá has one of the country’s most important lakes, Lake Atitlán, which is a primary economic resource, as it is central to a commercial route and is a key tourist attraction [20]. Its population of 421, 583 people is composed mostly of indigenous Mayans (96.4%). Sololá has a large percentage of Tz’utujil (61.1%), Kaqchikel (14.8%), and K’iche’ (10.7%) speakers [16].

### Study design

For the needs assessment, we identified levels of the MoH most relevant for the program: central-level SIAS and the first and second levels of care. Within each level, we used purposive sampling to identify a range of actors familiar with different aspects of hypertension prevention and care within the system. We began by interviewing national-level Ministry of Health staff and administrators and health system experts from prominent institutes focused on health system practice and research—we refer to this group as national-level health system stakeholders for the remainder of the article. National-level health system stakeholders provided insights about the management, system level needs and considerations for the intervention. Then we used purposive sampling to identify a similar set of participants at the health area, health district and community levels in the two departments/provinces (Zacapa and Sololá). At the provincial level, we interviewed MoH Area directors, staff familiar with chronic disease management and team members responsible for medication supply. At the health district level, we spoke with health district directors, doctors, professional nurses, and auxiliary nurses that work at the health center and health post levels. At the community level we spoke with hypertensive patients (male and female adults), family members and community level health experts (traditional birth attendants and healers), and community leaders.

We used semi-structured interview guides to conduct interviews and focus-group discussions (FGDs).^2^ Interview guides included questions about the health care system building blocks, local perceptions of hypertension, and implications for the hypertension control intervention. FGDs with auxiliary nurses explored their working relationship with the community, service provision for patients with hypertension, teamwork, use of MoH treatment guidelines, training, and thoughts about the proposed intervention. FGDs with patients explored their thoughts about health, hypertension management, care-seeking behavior outside of MoH facilities, access to medications, and thoughts about the proposed intervention. No FGDs were conducted with national-level health system stakeholders because it was more appropriate and feasible to conduct interviews with them in order to capture their unique experiences and knowledge about the health system.

### Participants

Two members of our team, SC and JCF –a psychologist and an anthropologist both experienced in qualitative research– began data collection with national-level health system stakeholders from March to May 2018 in Guatemala City. Interviews with national-level health system stakeholders were held at MoH buildings as well as non-governmental organization offices. These participants were knowledgeable about Guatemala’s public health care system.

**Fig. 1:**
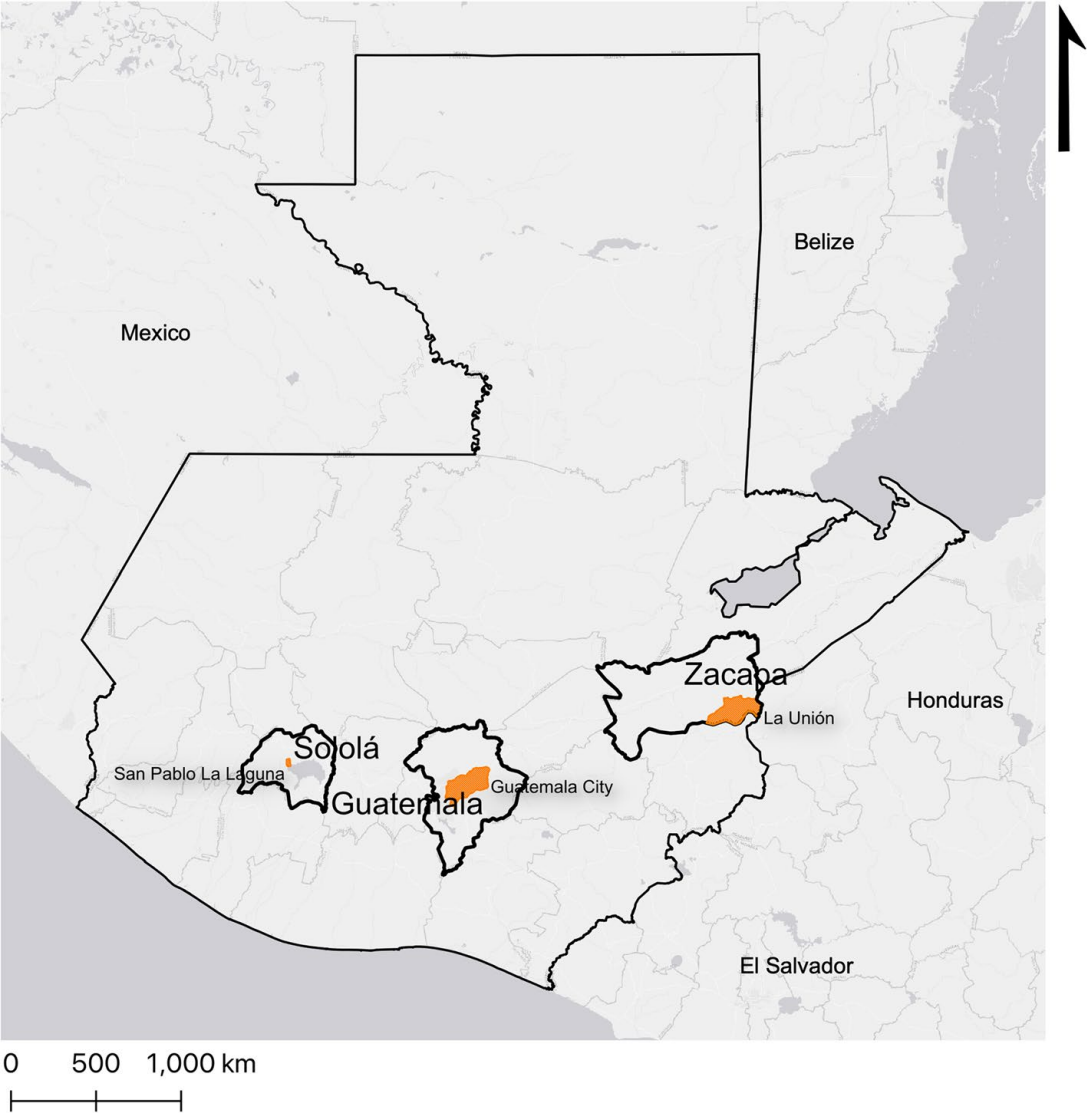
Map showing the three locations of the study at the department and health district level. Data source: Infraestructura de Datos Espaciales de Guatemala; Open Street Map; Map source: ArcGIS Online map hosted by ESRI

Then, in June 2018, we conducted individual interviews and FGDs with participants from different levels in Zacapa and Sololá, two of the five selected departments of the implementation study. These departments correspond to two MoH health areas where, within each of them, we selected a MoH health district: La Unión for Zacapa and San Pablo la Laguna for Sololá (Fig. 1), where we conducted interviews with health providers and administrators, patients, family members and community members, as well as FGDs with auxiliary nurses and patients.

### Procedures

We conducted interviews with patients, family members and community members at patients’ homes, community spaces, and in MoH facilities. We held FGDs with patients and auxiliary nurses at MoH facilities and community spaces. Staff from the health posts attended interviews and one FGD held in two Mayan languages (Kaqchikel and Tz’utujil) in the department of Sololá, in order to provide simultaneous translation to Spanish. We conducted interviews and FGDs until we reached data saturation when no new themes emerged.

For interviews, we followed a purposive sampling approach. We reached out to potential national-level health system stakeholders, and health providers and administrators who fulfilled the purposive sampling attributes via e-mail and/or phone. We approached patients and family members who had been identified by MOH staff at health posts and health centers face-to-face. We later made home visits; during most of the interviews with patients, one or two MOH auxiliary nurses were present because they led the way to the patient’s home and/or helped translate between Spanish and Mayan languages. For FGDs with patients, MOH staff at health posts and centers invited hypertensive patient participants. For FGDs with auxiliary nurses, MOH health providers and administrators made the invitations to participate. All interviews and FGDs were audio-recorded and fieldnotes were taken during and afterward.

Interviews with family members lasted from 10 to 20 min; with patients, 20 to 50 min; health staff, 20 to 50 min and national-level health system stakeholders, 30 to 90 min. FGDs with patients lasted from 70 to 95 min; with auxiliary nurses, 90 to 110 min. Time with each participant type varied due to topics in the specific interview and FGD guides, and was also based on their availability. We did not carry out follow-up interviews.

Although we analyzed interview transcripts with health staff at the health area level, community level health experts, and community leaders, we did not include them in this article because the information they provided was limited regarding the explanatory models of illness framework.

This study was approved by INCAP’s Institutional Review Board (IRB) and the Guatemalan National Health Ethics Committee. In addition, IRBs from the Tulane University School of Public Health and Tropical Medicine, the Institute for Clinical Effectiveness and Health Policy in Argentina and the Colorado School of Public Health approved the study. All participants provided written informed consent.

### Analysis

JCF transcribed all interviews and FGD recordings in Spanish. A Kaqchikel-speaking member of the study team (ISC) translated and transcribed patient and family member interviews conducted in Kaqchikel for linguistic reliability. As part of the first phase of analysis, two study team members (JCF and SC) coded transcripts with Nvivo 11 Pro. We applied framework analysis, with pre-defined codes in Nvivo based on Kleinman’s explanatory model domains. Specific codes that we used from Kleinman’s explanatory model domains [14], were: etiology, symptoms, pathophysiology, course of illness, and treatment. Following are the specific code definitions that we used. We defined etiology as the causes of hypertension, including social, emotional, lifestyle, and biological causes. We defined symptoms as onset and presence of symptoms caused by hypertension: any sensation, feelings, or changes in the body. Pathophysiology was understood to be the mechanisms and effects of hypertension in the body from a biomedical standpoint –factors such as: underlying renal or adrenal disease, salt intake, obesity, insulin resistance, renin-angiotensin system, sympathetic nervous system, genetics, endothelial dysfunction, low birth weight and intrauterine nutrition, and neurovascular anomalies [21]. We defined course of illness as the kind of sickness –acute or chronic, curable or not, its temporality and severity, and how it affects the patient’s life. We defined treatment as any biomedical, natural, and traditional treatment, and lifestyle and behaviors related to hypertension care.

In a second phase of analysis, we went back to the transcripts to identify emerging concepts and specific quotes, and used matrix analysis [22] to compare the five Kleinman’s explanatory model domains for different types of participants. Matrix analysis is a way to cross dimensions in rows and columns to look for patterns and make comparisons [22]; we conducted this analysis for explanatory model dimensions by type of participants. We prepared reports for each type of actor, and included a summary of findings and exemplary quotes that captured recurring themes. With these summaries we elaborated a final matrix, which is our Table 2, and selected quotes for the text.

Our study team held regular meetings to discuss the process of analysis for the first and second phases. In the first phase, we defined the definition of each code and what was to be coded. During sessions, we discussed and came to agreement on the clarity of code definitions and the adequacy of coded segments, which led to redefining codes and recoding sections. In the second phase of the analysis, we focused on the contents of the matrix (quotes) and themes, and came to agreement on how to summarize and present results.

## Results

In Table 1 we summarize the number of interviews and FGDs by location that we included in the analysis. We analyzed interviews for 61 people from Guatemala City, Zacapa and Sololá. We analyzed six focus group discussions: three with auxiliary nurses and three with patients.

**Table 1:** Different types of participants who took part in interviews and FGDs by location

**Type of participant**	**Interviewed**	**Focus-group discussions**	**Location**
National-level health system stakeholders	17	–	Guatemala City
Health providers and administrators	13	2 (11 & 6 auxiliary nurses)	Zacapa
	12	1 (9 auxiliary nurses)	Sololá
Patients and family members	10	2 (9 & 4 patients)	Zacapa
	9	1 (8 patients)	Sololá
**Total**	**61**	**6 (47 participants)**	

In Table 2, we summarize the results from the matrix-based analysis, including data from interviews and FGDs. Table 2 compares explanatory model domains –etiology, symptoms, pathophysiology, course of illness and treatment– for the three different actor types: patients and family members, health providers and administrators, and national-level health system stakeholders.

**Table 2:** Explanatory model domains by actor type

**Explanatory Model Domains**	**Patients and family members**	**Health providers and administrators**	**National-level health system stakeholders**
**Etiology** (causes of hypertension)	It’s inherited, comes from sadness, anger, and worry	It comes from old age, anger, worry, a sedentary life	Hypertension comes from consumption of unhealthy food or energetic causes
**Symptoms** (description of sensations, feelings, and physical changes)	Causes pain in the body, headaches, nerves, weakness	Causes pressure in the chest, pain in the body, headaches	Patients don’t identify its symptoms, it’s silent. Symptoms from MoH Primary Health Care Guidelines (headache, blurry vision, dizziness, pressure in the chest, and ringing in the ear)
**Pathophysiology** (physiological mechanisms and their measurability)	Caused by salt intake, stress, is inherited, co-occurs with diabetes	High blood pressure is hard to identify and treat using standard parameters (≥ 140/90 mmHg)^a^; some patients feel normal with high blood pressure	Identification derives from what the MoH Primary Health Care Guidelines identify as hypertension
**Course of illness** (kind of disease –chronic or otherwise–, temporality, severity)	Because of its chronic quality, it needs daily attention, but it is hard to comply with recommendations (diet, medication, exercise)	When patients feel better, they stop the treatment or forget to take it. Patients think that having hypertension is normal	Patients take medicines but don’t follow lifestyle recommendations. They think having hypertension is normal. Patients don’t have enough information about hypertension
**Treatment** (medical and traditional, habits and behaviors that improve the patients’ condition)	Taking hypertensive medicine is worrisome because of its long-term consequences and secondary effects (such as vomiting). Patients use natural medicine, store-bought, pharmacy, and health post medication when available. Being calm is a way to feel better	Patients self-diagnose and take natural medicines. When available, patients take medicines given at health posts	According to MOH Primary Health Care Guidelines, hypertension should be treated at the primary health care level, but there is no regular medication availability, not enough trained staff, and no medical tools to detect it and treat it properly

^a^During data collection, Guidelines defined hypertension in two stages: (1) SBP 140–159, DBP 90–99; (2) SBP higher than 160, DBP higher than 100 mmHg [23]. Nowadays, Guidelines define hypertension as SBP > 130, DBP > 80 [24]

### Explanatory model of illness domains

In the following paragraphs, we present each explanatory model domain along with illustrative quotes from participants representing different perspectives.

### Etiology

Most patients referred to hypertension as being caused by emotional distress or they viewed it as being inherited. For example, a patient from a FGD related the moment she was diagnosed with hypertension during her grieving process after she lost her son. She explained the link to emotional distress through an auxiliary nurse who translated to Spanish:

*“It happened when her son passed away. For a long time, she was sad, she cried and was agitated. That is when they told her she had high blood pressure, because of all the mourning process she had been through.”* (FGD with patients, San Pablo La Laguna; Tz’utujil-speaking patient).

In contrast, some national-level health system stakeholders described that hypertension is mainly caused by unhealthy eating habits. As an example, this participant linked current junk food availability with increasing rates of hypertension, suggesting that the past was healthier:

*“I can speak about rural areas where bagged snacks, sodas, instant soups, and junk food were not available. Today they are children and people’s daily bread. The way they see life has changed; now it is about having material possessions. Before, it was about nurture, living peacefully, being well.”* (National-level health system stakeholder).

Another cause mentioned by national-level health system stakeholders was related to energy, which is something beyond a doctor’s [biomedical] comprehension. Health providers and administrators (doctors and nurses) referred to both explanatory models (patient and national-level health system stakeholder), acknowledging emotional causes like anger, and lifestyle causes like being sedentary.

### Symptoms

In general, hypertensive patients shared that they mainly feel headaches and most also described feeling tired and weak. This is captured by a patient from a FGD who described feeling a headache as a sensation of fire and pain; she also mentioned feeling something in her stomach, explaining what she feels as a ball, but making it seem like something that is almost indescribable:

*“My head gets like it’s on fire; those pains grab me here behind my head. Something gets on top of my stomach, like a ball in the tip of my stomach.”* (FGD with patients, La Unión).

Health providers and administrators, mostly auxiliary nurses, coincided with the symptoms communicated by patients and pointed out that patients may seem debilitated and dizzy. National-level health system stakeholders argued that patients in general won’t perceive symptoms, explaining that hypertension is silent, or in other words, that having the disease is not the same as feeling it. as described in this quote:

*“What must be done, in the first place, is to improve hypertension detection. Because, as you know, half of people with hypertension don’t know they are hypertensive; they don’t have symptoms.”* (National-level health system stakeholder).

### Pathophysiology

Patients diagnosed with hypertension in the study described how it feels and were able to point out some of the risk factors known to be part of the pathophysiology of hypertension: salt intake, inheritance, co-occurrence with diabetes, and stress. Health providers and administrators and national-level health system stakeholders referred to standardized procedures that they put into practice. In that sense, they perceived the pathophysiology of hypertension as directly tied to what the MoH Primary Health Care Guidelines state.^3^ This is exemplified in the linear process of carrying out a physical evaluation as a means of categorizing and then treating a patient, as a national-level health system stakeholder explained:

*“First, a clinical evaluation is done for the patient, blood pressure is measured. Here the guidelines indicate how to classify it and depending on that, adequate treatment is given, always according to the guidelines.”* (National-level health system stakeholder).

Knowledge is based on standardized measurements that are also used in other biomedical contexts, like defining the Ministry of Health guidelines according to the US National Heart, Lung and Blood Institute-appointed Joint National Committee (JNC) guidelines, as the next participant explains:

*“The guidelines are based on the 8*^*th*^* JNC report, the hypertension report from the United States. So those are the ranges we use, pre-hypertension stage 1, stage 2…”* (National-level health system stakeholder).

However, health providers and administrators in La Unión, Zacapa explained that they found it difficult to rely solely on standardized procedures to diagnose and treat hypertension. Several of them pointed out that some patients may feel okay with measurements that signal hypertension:

“*I have seen people who, for example have 140/90, people that feel normal, that feel fine with their blood pressure. However, we may find people with that blood pressure that can be dying.*” (Auxiliary nurse, La Unión).

### Course of illness

For patients in this study, technical aspects of hypertension (such as pathophysiology) were not understood in detail. Some saw hypertension as a temporary condition, while others referred to it as being permanent. Most patients in the study shared that they have found it difficult to comply with treatment. Most family members explained that patients usually stop their treatment when they feel better and may take it again when they feel sick. They described a similar behavior for health post visits. For those in treatment, a good number of patients said that they did not keep appointment dates defined by health providers and administrators and sought care when they felt sick. Most patients also stated that it was difficult to adhere to diet and exercise changes. Some explained that they had been able to reduce salt and sugar consumption, but that was not always the case. Others mentioned not having time to do exercise or that the concept of exercise was unfamiliar. The case of the next patient is an example of how it may be difficult to comply with diet because of the values attached to her role in the community. She explained that as a *comadrona* [midwife], she has found it necessary to accept the many offerings of sweetened drinks, coffee and food from her patients; but she was able to comply with the exercise recommendations:

*“It’s hard to maintain my diet. Because of my job I visit a lot of homes, people offer me food, coffee, pinol with a lot of sugar. I can’t reject it because it would be frowned upon. I have resigned myself; I can only walk and do exercise because with food, I can’t.”* (Kaqchikel-speaking patient, San Pablo La Laguna).

In a few cases we found patients or family members who referred to being able to manage their blood pressure by having a blood pressure monitor:

*“I had to buy a monitor and check it [family member’s blood pressure] myself when she tells me ‘look, I’m feeling something’.”* (Family member, La Unión).

For financial reasons, not all patients were able to afford a blood pressure monitor.

From the perspective of most national-level health system stakeholders, they viewed patients as having a limited understanding of hypertension and a low level of hypertension management due to disinterest in taking care of themselves. Many stated that patients normalize living with untreated hypertension and make minimum effort – just taking medicine but not making other changes –, as the next comment exemplifies:

*“The problem with hypertensive patients is that –I say it from my experience– they are hypertensive because somehow, they see it as …part of daily life. They limit themselves to taking some antihypertensive medication and don’t even diet….”* (National-level health system stakeholder).

On the other hand, national-level health system stakeholders recognized that they lack capacity and resources to provide education and information about hypertension to patients.

Health providers and administrators’ perspectives of hypertension are closer to patients than national-level health system stakeholders; they recognize difficulties and treatment compliance in a similar way to patients. They shared that some patients don’t take having hypertension seriously, but most worry about having hypertension and follow indications as best as they can –sometimes perceiving it as a severe harm to their lives; they also pointed out that it is harder for older patients to follow recommendations.

### Treatment

Most of the medication mentioned by patients are treatments for hypertension, enalapril being the most common, but also captopril and atenolol. Nevertheless, they also mentioned medication for diabetes (metformin and glimepiride), analgesics (acetaminophen, diclofenac) and others commonly used to “calm nerves” like B-complex vitamins. Patients also referred to using natural remedies such as orange, lemon, grapefruit and soursop leaf teas, chamomile, and garlic. In relation to diet and lifestyle, some patients reduced coffee, salt, and condiment consumption, and increased water intake and exercise. Patients also mentioned trying to stay calm and reduce stress. The next patient mentioned using treatments other than medications from health posts or pharmacies:

*“When I got sick, they told me to stop everything: salt, coffee, candies. Someone then recommended I drink electrolyte solution and lemon and orange leaf tea. Also, they told me that water was what I needed most. Now I don’t take pills.”* (Kaqchikel-speaking patient, San Pablo La Laguna).

In addition to medicines from health posts (typically enalapril), health providers and administrators also recommended natural remedies at times, such as those mentioned by patients, and recommended changes to diet and lifestyle (reduced salt consumption, healthy diet, and regular exercise). In some cases, local MOH health providers and administrators would visit patients at their homes to support them.

Some health providers and administrators, and national-level health system stakeholders, interviewed in this study spoke about treatment based on MoH guidelines as demonstrated by this next quote:

*“Right now in the [MoH] facilities we have: hydrochlorothiazide, enalapril, and losartan. Those three medications are on the basic list. Others are for hospital use, the third level [of care].”* (National-level health system stakeholder).

However, in our needs assessment we found that not all of the medications from the guidelines were available at health posts. Another important barrier that participants mentioned was hypertension detection, which has been a challenge due to limited training on the topic for health care providers and lack of monitors at the primary health care level.

### Cross-cutting themes

We identified three themes that were relevant and cross-cutting for various explanatory model domains. These were: symptoms and detection; treatment/management preferences and access; and communication***.***

### Symptoms and detection

Patients in this study explained that symptoms of hypertension initially became apparent because they could feel them. Sometimes these symptoms were something new or different, but in some cases they were common, not generating any concern. This meant that hypertension was typically identified when a patient felt symptoms but was not diagnosed until after seeking care at a health post or center. In some cases, a patient had symptoms from a different disease, went to a health facility, and hypertension was detected by chance. A national-level health system stakeholder explained that when people go to a health facility with a common condition, their hypertension would often be missed:

*“I’ll try to explain: I [referring to being an average patient] go to a health facility with the flu; they will treat me for the flu, but they won’t check me comprehensively. They won’t check my risk factors: if I smoke, if I’m overweight, if I have a bad diet, if I don’t do exercise, if I consume alcohol in excess; I mean, they don’t check risk factors that may lead me to having [hypertension].”* (National-level health system stakeholder).

Care at many facilities in the public health system may not detect hypertension as many patients go to a health facility not knowing what they have and only report symptoms that they feel.

### Treatment/management preferences and access

Before seeking care at a health facility, and when feeling symptoms of hypertension, most patients referred to seeking natural remedies and/or medication sold at stores and pharmacies. When patients described seeking care at a facility, they only received medication in some cases. But most patients reported not relying on medicine from the public health system because it is not regularly available. In these cases, only a prescription is given out, but patients may not be able to afford the medicine, as an auxiliary nurse in a FGD described:

*“Sometimes patients don’t have money. They come to ask, and one doesn’t have [medicine]. That is a difficulty for treatment. In those cases, as other nurses said, we give them natural treatments. That helps them keep their blood pressure stable while they get the money to buy treatment.”* (FGD with auxiliary nurses, La Unión).

Non-pharmaceutical treatments, such as natural remedies, are sometimes recommended by health providers and administrators when medicine is not available at Ministry of Health facilities and when patients can’t afford to purchase medicines at a pharmacy. Some patients mentioned that pharmaceutical treatment received at the health post provoked side effects, which is another reason they sought natural remedies.

Health providers and administrators also recommended lifestyle changes (such as diet and exercise) but not all patients were able to comply with them. Some patients -especially in rural areas- did not have a concept of exercise, as a patient from a rural area explained:

“*I don’t know how to do exercise. I don’t have time and I don’t know what it is. I only walk.*” (Kaqchikel-speaking patient, San Pablo La Laguna).

Patients also shared that they may not be able to buy recommended foods and/or it may not be available where they live.

Patients that perceived hypertension as something temporary might prefer to continue their life without introducing changes in diet, exercise and medication. Other patients referred to making partial changes (e.g. sometimes, until they feel better) so they would not get used to medication, because they were tired of taking it, or due to fatalistic views (e.g. “they have to die of something”). One participant explained trying natural medicine after being tired of taking large quantities of pills and also feeling a sense of desperation:

*“My late mother took me to a natural medicine provider to try natural medicine because I was tired of taking so many pills. I resigned myself to the fact that if I died, oh well… At the end, everyone passes away one day and that includes me. I was desperate.”* (Kaqchikel-speaking patient, San Pablo La Laguna).

### Communication

Health providers and administrators shared that at times it may be difficult to communicate how hypertension affects a patient’s health and how it should be treated. Most patients did not have a clear idea of what hypertension is and the extent to which it is a chronic condition. For some patients, the chronic nature of hypertension is a shift in thinking as they are accustomed to conditions that are cured with taking medicine on a temporary basis. In some cases, communication about hypertension may be confusing if they also have another condition such as diabetes, as explained by this patient:

*“After some time, I went back to the health center because I got worse. When I got there, they told me my sugar was at 600 and that I had diabetes. That’s what I don’t understand. ¿Why did they tell me it was hypertension, and then diabetes?”* (Kaqchikel- speaking patient, San Pablo La Laguna).

Difficulties may also arise when patients speak languages other than Spanish, such as Kaqchikel and Tz’utujil, the languages spoken by most patients interviewed in Sololá. Health providers and administrators recognized the importance of giving explanations using the local language. In some cases, communication may be facilitated when health providers and administrators are from the same communities as their patients and speak the local language(s). This is more common for auxiliary nurses as explained below:

*“…It’s all about the doctor’s strategy... sometimes they take us –auxiliary nurses–, who can explain in the person’s language what to do.”* (Auxiliary nurse, San Pablo La Laguna)

Knowledge of the local non-Spanish language is not always the case –particularly with health providers and administrators higher up in the hierarchical structure and in larger urban centers.

Additionally, health education and other informational resources are mostly available in Spanish, which can discourage patients from seeking information, such as this patient who referred to the importance of offering information in a patient’s first language:

*“Most of the people that can and want to participate in these informational sessions don’t speak Spanish and don’t understand much about what is being talked about. People get desperate because they don’t understand. It has to be in Kaqchikel.”* (Patient, San Pablo La Laguna).

## Discussion

Following Kleinman’s explanatory model [14], we were able to organize stakeholders’ understandings about hypertension in a comprehensive way, comparing the same domain between different actors in the public health system. We believe this approach shows in detail how patients’ explanatory models are sometimes similar but often different from those of local-level health providers and administrators, and national-level health system stakeholders. The diverse ways of understanding hypertension and how to prevent and treat it are important to take into consideration and present an opportunity for improving communication and delivery of health services.

Patients, family members, and health providers and administrators referred to the causes of hypertension as being related to emotions, inheritance, and age, which is similar to what has been reported in other studies [25, 26]. National-level health system stakeholders put more weight on unhealthy habits, such as consumption of unhealthy food. They did not interpret emotional distress in the same way as patients, who referred to a bad moment in their life as the cause of hypertension. In a way, national-level health system stakeholders put more weight on a cause that patients may control but that is also related to structural and environmental changes: eating or not eating healthy food, and broader food environments. Whereas patients and health providers and administrators put more weight on factors that are primarily outside the individual’s control: inheritance and age; as well as, management of emotions, which is a combination of internal factors and external stress. As national-level health system stakeholders put more emphasis on eating habits and the food environment, there may be an opportunity to get them engaged in initiatives focused on healthy eating at both the individual and built environmental levels.

Patients and local-level health providers and administrators referred to headaches and pain, weakness, and dizziness as the main symptoms of hypertension, which are in line with findings in other studies [25, 27]. In terms of defining what hypertension is, only national-level health system stakeholders and health providers and administrators were familiar with biomedical definitions, as they are in the guidelines they use to identify and treat hypertension. Patients had a limited understanding of the pathophysiology and other aspects of hypertension. This implies a distance between what patients know and understand and what health providers and administrators know and understand.

Health providers and administrators, and national-level health system stakeholders shared that patients often do not work to manage their blood pressure and stop taking medications. But by hearing from patients, we realize that the situation is more nuanced. Patients often do not have the financial means to take care of themselves (e.g. they are often not able to buy medicines or a blood pressure monitor) but also face difficulties related to the broader context in which they live and work (e.g. they have too much work, stressful living conditions, and limited healthy food availability). In our study, economic difficulties and access were impediments to management and care. Adherence to medication was described as a difficulty related to medication not being regularly available for free at health posts and health centers. A focus on addressing contextual challenges rather than relying on individual patients to overcome them, is important for addressing causes of hypertension. In order to support adherence, it will be key to secure access to free medications that are consistently available.

Some patients also shared that they have difficulty complying with treatment because they understand hypertension as a short-term condition (as compared to the biomedical perspective). The understanding of hypertension as a transient/temporary condition may explain why some patients abandon treatment after a while. In some cases, patients spoke about stopping treatment because of side effects, something also present in another study that examined explanatory models of hypertension [25].

National-level health system stakeholders referred to self-medication as a negative practice. Nevertheless, it is very common that patients seek treatment outside Ministry of Health settings in Guatemala, which is also common in other country contexts, and often includes the use of natural remedies [26, 27]. In our study, health providers and administrators sometimes recommended natural remedies. This shows that they are closer to knowing the reality of their health care facilities, which often lack a reliable supply of medicines, and they understand that patients have limited resources to purchase medicines elsewhere. They may be more open to alternatives. In that sense, they are an important link between patients and national-level health system stakeholders.

An important barrier for engaging patients in hypertension care is a gap in language and communication. This was especially referred to as a problem by people in Sololá; it is a challenge for patients who are most comfortable speaking a Mayan language such as Tz’utujil and Kakchiqel to understand the health providers and administrators, who usually speak Spanish. There is a gap in communication about what hypertension is and how it should be treated and managed. Language and communication gaps have been identified as a barrier for community engagement, which limits information sharing with communities about the importance of hypertension and reduces opportunities for health education, as is the case for a study in Ghana [28].

We recognize that an emphasis on hypertension must also consider other emerging chronic disease priorities in Guatemala. In a recent population-based survey of chronic kidney disease in two rural municipalities in Guatemala, the majority of cases were associated with hypertension [29]. Diabetes and hypertension are closely related and are often detected at the same time and need to be managed together. While our study focused specifically on hypertension, it is critical to understand perceptions around other chronic conditions, recognizing that many people will experience them together and that the health system aims to develop comprehensive approaches to address chronic disease.

### Limitations

This study had several important limitations. Due to this study being a secondary data analysis, interview guides were not elaborated specifically to capture each of the explanatory model domains and some perceptions about specific domains were surely missed. Nevertheless, the breadth of data gathered allowed for an analysis of the domains by type of participant.

The translations from Tz’utujil to Spanish were made by MOH staff who were present during interviews and FGDs. This is different from the interviews conducted in Kaqchikel, that were later translated by a member of our team (ISC) who is trilingual in Spanish, Kaqchikel and English. Some of the ideas from participants in interviews and FGDs conducted in Tz’utijul and simultaneously translated to Spanish may have been summarized for fluidity. As a result, we may have missed some of the nuance of ideas expressed in Tz’utijul.

While the study included a range of participants, it did not include the perspectives of local traditional healers. In the future it will be important to include traditional healers to help understand more about self-care practices of patients and the variety of options available for care in communities other than health posts and health centers. As we saw, natural remedies and medicines bought from pharmacies were commonly used by patients. In the future, we will be interested to explore how these therapeutic practices influence care and the role that traditional healers and other community providers play in addressing non-communicable diseases such as hypertension.

### Implications for the health system and strategies to encourage understanding

Considering what healing means in a health system, Kleinman refers to *cultural healing* with the implication that it is not only the health system but “the cultural system as a whole which heals” [14]. On that note and following what Kleinman considers the “core adaptive tasks of health systems” [14], we found some challenges to the health system being capable of *healing* and opportunities for improving understanding across different actors. Cultural meanings of what hypertension is varied by the understanding of hypertension, and was primarily understood in biomedical terms by providers and national-level actors. Strategies that guide choices for healing also varied, with patients using herbal remedies and medicines from stores and pharmacies which national-level health system stakeholders referred to as a negative behavior. Health providers and administrators referred to patients as not grasping the chronicity of the disease; however, the therapeutic interventions patients sought often went beyond those delivered by health providers and administrators. We consider that the perception of health-enhancing behaviors as being abandoned by patients must be understood in the context that health-enhancing interventions are often not offered or available to them; education and promotion of health behaviors, backed by a well-supplied and accessible health system could make an important difference in their behaviors.

Several strategies may allow actors who are situated differently in the system to understand each other more. Patient education may be an important activity for patients to learn more about hypertension, beyond risk factors which they referred to understanding. Intercultural exchanges that include traditional healers may be a way for different actors to express their understandings of health and disease [30]. Activities and exercises that more broadly recognize diverse mental models, or the representation of how some aspect of the world works, in this case, conceptual knowledge and beliefs, also may encourage understanding between different actors in a system [31].

An analytic approach such as the one we used in this article may be useful in improving understanding between different types of actors within a system. When thinking about removing barriers that prevent hypertension control, recognizing where different actor types are situated in the system may enhance approaches so that on the one hand intervention approaches invest in patient education and a shift toward recognizing hypertension as a chronic condition and on the other hand focus on investing resources to ensure a regular supply of medication.It is important to consider how system infrastructure and resources influence behaviour and identify actors who serve as bridges to improving understanding. Our application of the Kleinman model to hypertension involving different types of actors within the system could be used for other conditions in Guatemala and may be applied elsewhere in Latin America and globally.

## Conclusion

As perceptions and understandings of hypertension vary for different actors, it is important to describe their similarities and differences considering the role each one has in the health system and the possibilities that those understandings may bring when designing and improving hypertension management programs. The Explanatory Models Framework domains provides a way to understand these diverse perspectives in depth. With respect to etiology, patients and health providers and administrators spoke about the causes of hypertension as being related to emotions, inheritance, and age, while national-level health system stakeholders focused on unhealthy habits. For patients and health providers and administrators, symptoms had to do with feeling pain and headaches, whereas national-level health system stakeholders spoke about symptoms as hard to identify or not relevant for hypertension. Health providers and administrators and national-level health system stakeholders referred to patients as making limited effort to manage their blood pressure, but did not always put this into the context of the challenges that patients face in trying to access treatment. From the perspective of patients, they cannot rely on the health system because medication is not always available and they often seek treatment outside of a public health facility. More emphasis should be put on context and availability of resources (such as access to healthy food, language interpretation, and socio-economic status in communities) which could make efforts at healing more feasible and accessible.

## Data Availability

The datasets generated and analyzed during the current study are not publicly available to protect participants’ identities and because the type of consent received from participants does not allow it; but de-identified data are available from the corresponding author on reasonable request.

## Abbreviations

LMICsL: Low and middle-income countries
HICs: High-income countries
MoH: Ministry of Health
SIAS: Sistema Integral de Atención en Salud (English translation: Comprehensive Health Care System)
WHO: World Health Organization
INCAP: Instituto de Nutrición de Centro América y Panamá (English translation: Institute of Nutrition of Central America and Panama)
FGD: Focus-group discussion
JNC: Joint National Committee

## Footnotes

1 Ladino is a term originating from colonial times that was used for people who were neither Spanish nor indigenous. Over time, the term has been modified to become a way to define a “general mestizo” [32] and is considered an ethnic group.

2 Our guide for interviews and FGDs was first published and can be found in the Supplementary Information section of the Fort, M., et al. (2021) [12] article.

3 The MoH Primary Health Care Guidelines state as risk factors: age (more than 65 years old), sex (more frequent in women), family history, smoking, sedentary lifestyle, high salt intake, obesity, high cholesterol, diabetes, and renal disease; as symptoms: headache, blurry vision, dizziness, chest tightness, and ringing in the ears; and can start with: lethargy or unconsciousness, excessive sweating, seizures, edema, and nose bleeding [23].
